# Supplementary material for: The Combining Ability and Heterosis Analysis of Sweet–Waxy Corn Hybrids for Yield-Related Traits and Carotenoids
Source: Plants (Basel). 2024 Jan 18;13(2):296. doi: 10.3390/plants13020296 (PMC10819934; doi:10.3390/plants13020296)
Supplement: Supplementary file 1 [file plants-13-00296-s001.zip › plants-2810853-supplementary.pdf]

Table S1 Mean performances of parents, hybrids, and commercial checks for agronomic traits across two seasons between 2021 and 2022.

Genotype	Hey (ton/ha)	Hed (cm)	Hel (cm)	WSS (°Brix)	Ph (cm)	Hd (DAP)
Parents						
ILS_1	6.82	3.64	12.18	12.66	136.00	62.75
ILS_2	10.49	3.72	14.36	14.64	136.25	62.38
ILS_3	10.33	4.00	14.23	12.76	130.50	62.50
ILS_4	14.97	4.10	18.01	13.65	193.50	62.88
ILS_5	10.44	3.87	15.38	12.95	136.75	63.75
ILS_6	10.44	4.62	15.14	14.34	139.75	62.75
ILS_7	8.52	3.97	15.04	12.75	129.00	61.75
ILS_8	4.32	4.01	12.90	13.35	129.25	60.63
ILW_1	6.16	3.05	12.70	11.95	149.25	60.50
ILW_2	6.51	3.31	13.13	10.61	133.75	59.00
ILW_3	9.99	3.63	14.84	10.20	136.50	58.63
Hybrids						
$ILS_1 \times ILW_1$	21.27	4.73	19.39	11.79	203.17	58.67
$ILS_1 \times ILW_2$	16.53	4.34	17.53	11.51	174.67	56.42
$ILS_1 \times ILW_3$	21.06	4.62	19.06	10.97	193.83	57.17
$ILS_2 \times ILW_1$	25.37	4.62	20.28	11.99	209.50	58.58
$ILS_2 \times ILW_2$	19.03	4.27	18.68	11.72	171.00	56.92
$ILS_2 \times ILW_3$	18.49	4.60	18.08	11.66	189.50	57.33
$ILS_3 \times ILW_1$	20.71	4.74	19.13	10.95	191.83	60.25
$ILS_3 \times ILW_2$	17.94	4.42	19.10	10.45	173.17	58.33
$ILS_3 \times ILW_3$	20.30	4.83	17.98	10.85	176.83	58.83
$ILS_4 \times ILW_1$	21.24	4.71	20.75	11.45	216.25	57.17
$ILS_4 \times ILW_2$	20.43	4.40	20.57	11.28	185.50	57.17
$ILS_4 \times ILW_3$	21.91	4.74	19.39	11.27	200.83	56.92
$ILS_5 \times ILW_1$	21.37	4.65	20.29	12.25	194.67	60.00
$ILS_5 \times ILW_2$	18.77	4.34	20.27	10.86	172.50	58.42
$ILS_5 \times ILW_3$	20.23	4.71	19.44	11.07	185.33	58.58
$ILS_6 \times ILW_1$	14.25	4.58	18.80	10.21	196.67	56.33
$ILS_6 \times ILW_2$	20.38	4.60	19.03	10.98	179.50	56.33
$ILS_6 \times ILW_3$	20.22	4.90	18.22	11.04	184.00	57.45
$ILS_7 \times ILW_1$	21.35	4.89	20.43	11.10	202.00	57.17
$ILS_7 \times ILW_2$	21.05	4.49	18.77	10.76	173.33	56.83
$ILS_7 \times ILW_3$	20.04	4.84	18.29	11.36	182.00	58.00
$ILS_8 \times ILW_1$	18.66	4.82	18.28	11.22	211.33	56.58
$ILS_8 \times ILW_2$	17.93	4.57	17.10	11.60	163.67	56.00
$ILS_8 \times ILW_3$	19.10	4.74	17.39	11.18	172.00	56.50
Mean of females	9.54	3.99	14.66	13.39	141.38	62.42
Mean of males	7.55	3.33	13.56	10.92	139.83	59.38
Mean of hybrids	19.90	4.63	19.01	11.23	187.63	57.58
Mean of top five hybrids	22.25	4.86	20.46	11.88	170.47	56.32
Check 1	22.01	4.87	19.74	13.36	212.17	60.25

Genotype	Hey (ton/ha)	Hed (cm)	Hel (cm)	WSS (°Brix)	Ph (cm)	Hd (DAP)
Check 2	17.92	4.53	16.87	10.81	200.83	55.17
Mean of checks	19.97	4.70	18.31	12.09	206.47	57.71
LSD $_{0.05}$ (parents)	0.46	0.08	0.37	0.24	3.02	0.70
LSD $_{0.05}$ (hybrids and checks)	1.01	0.13	0.72	0.42	7.57	0.68

Hey husked ear yield; Hed husked ear diameter; Hel husked ear length; WSS water soluble solid; Ph plant height; Hd harvest date; DAP days after pollination.

Table S2 Mean performances of parents, hybrids, and commercial checks for carotenoids across two seasons between 2021 and 2022.

Entries	Carotenoid content (μg/g of FW)							β-Car/β-Cry	β-Cry/Zea	β-Car/(β-Cry+ Zea)
	TCC [1/]	Lut	Zea	β-Car	β-Cry	α-Xan	β-Cry+Zea			
Parents										
ILS_1	5.75	1.12	3.35	0.88	0.91	4.47	4.24	1.20	0.25	0.39
ILS_2	4.82	0.82	2.14	0.41	1.08	2.95	2.54	0.34	0.14	0.74
ILS_3	7.07	1.73	2.18	0.82	1.1	3.91	3.00	0.87	0.40	1.22
ILS_4	5.89	1.68	1.76	0.8	1.02	3.45	2.56	0.93	0.44	1.37
ILS_5	8.46	1.82	2.18	0.92	0.84	4.00	3.11	1.32	0.37	0.60
ILS_6	6.54	1.70	2.71	1.16	0.80	4.41	3.88	1.96	0.26	0.23
ILS_7	7.68	2.48	3.28	1.32	0.64	5.75	4.59	2.27	0.24	0.14
ILS_8	5.97	1.43	1.91	0.53	0.73	3.34	2.44	0.63	0.09	0.41
ILW_1	6.75	2.02	2.82	1.14	0.72	4.84	3.96	1.68	0.30	0.28
ILW_2	5.56	2.03	1.75	0.70	0.68	3.77	2.45	1.06	0.15	0.11
ILW_3	3.91	1.39	1.37	0.84	0.73	2.76	2.20	1.38	0.42	0.75
Hybrids										
ILS_1×ILW_1	7.15	1.33	4.16	1.23	0.27	5.49	5.39	4.64	0.23	0.06
ILS_1×ILW_2	6.09	2.71	1.28	0.77	0.57	3.99	2.04	1.42	0.39	0.51
ILS_1×ILW_3	4.56	1.24	1.85	0.99	0.25	3.09	2.83	3.95	0.36	0.15
ILS_2×ILW_1	7.57	1.50	4.67	1.37	0.39	6.17	6.04	3.67	0.23	0.08
ILS_2×ILW_2	5.80	2.62	1.38	0.81	0.46	3.99	2.19	1.89	0.37	0.34
ILS_2×ILW_3	5.35	1.36	1.77	0.98	0.27	3.14	2.76	3.67	0.36	0.15
ILS_3×ILW_1	7.28	2.62	2.61	1.26	0.21	5.23	3.86	5.94	0.33	0.08
ILS_3×ILW_2	5.53	0.73	3.24	0.71	1.39	3.96	1.44	0.58	0.53	1.97
ILS_3×ILW_3	4.20	1.73	1.03	0.89	0.77	2.76	1.91	1.18	0.47	0.75
ILS_4×ILW_1	6.12	2.24	2.61	1.05	0.23	4.85	3.66	4.71	0.29	0.09
ILS_4×ILW_2	5.16	3.40	0.82	0.73	0.78	4.22	1.56	0.94	0.51	1.30
ILS_4×ILW_3	4.04	1.34	1.17	0.99	0.40	2.51	2.15	2.48	0.46	0.35
ILS_5×ILW_1	5.30	2.25	2.09	0.93	0.22	4.33	3.01	4.21	0.31	0.11

Entries	Carotenoid content (μg/g of FW)							β-Car/β-Cry	β-Cry/Zea	β-Car/(β-Cry+ Zea)
	TCC [1/]	Lut	Zea	β-Car	β-Cry	α-Xan	β-Cry+Zea			
$ILS_5 \times ILW_2$	5.32	3.41	0.73	0.75	0.96	4.14	1.47	0.85	0.51	1.32
$ILS_5 \times ILW_3$	4.43	1.97	1.20	0.88	0.78	3.16	2.08	1.20	0.43	0.73
$ILS_6 \times ILW_1$	5.78	1.80	2.40	1.05	0.77	4.20	3.44	4.42	0.36	0.81
$ILS_6 \times ILW_2$	7.27	3.79	1.53	0.96	0.88	5.32	2.49	1.10	0.39	0.58
$ILS_6 \times ILW_3$	5.61	1.38	1.95	1.19	0.27	3.33	3.14	4.64	0.39	0.14
$ILS_7 \times ILW_1$	7.23	1.72	4.31	1.32	0.31	6.03	5.63	4.33	0.24	0.07
$ILS_7 \times ILW_2$	6.82	3.65	1.53	0.89	0.48	5.18	2.42	1.87	0.37	0.32
$ILS_7 \times ILW_3$	5.83	1.49	1.97	1.30	0.24	3.46	3.27	5.57	0.40	0.12
$ILS_8 \times ILW_1$	6.92	1.54	4.27	0.99	0.28	5.81	5.26	3.75	0.19	0.07
$ILS_8 \times ILW_2$	4.58	2.83	0.98	0.73	0.49	3.81	1.71	1.54	0.43	0.53
$ILS_8 \times ILW_3$	3.28	0.79	1.37	0.79	0.25	2.15	2.16	3.15	0.37	0.19
Mean of females	6.52	1.60	2.44	0.86	0.89	4.04	3.30	1.19	0.27	0.64
Mean of males	5.41	1.81	1.98	0.89	0.71	3.79	2.87	1.37	0.29	0.38
Mean of hybrids	5.72	2.16	2.02	0.98	0.50	4.18	3.00	2.99	0.37	0.45
Mean of top 5 hybrids	7.30	3.49	4.00	1.29	0.96	5.76	5.24	5.10	0.49	1.23
Check 1	3.66	0.82	1.82	0.80	0.76	2.64	2.62	1.43	0.37	0.89
Check 2	0.41	nd	nd	nd	nd	nd	nd	nd	nd	nd
$LSD_{0.05}$ (Parents)	1.62	1.00	1.24	0.35	0.30	1.35	1.53	0.78	0.10	0.36
$LSD_{0.05}$ (Hybrids & Checks)	0.10	0.07	0.06	0.02	0.04	0.10	0.07	0.20	8.98×10^{-3}	0.04

[1/] TCC total carotenoid content; Lut lutein; Zea zeaxanthin; β-Car β-carotene; β-Cry β-cryptoxanthin; α-Xan α-xanthophyll.
nd not detected.

Table S3 Specific combining ability (SCA) for yield-related traits of sweet-waxy corn hybrids evaluated across two seasons between 2021 and 2022.

Hybrids	Hey	Hed	Hel	WSS	Ph	Hd
$ILS_1 \times ILW_1$	1.03**	0.08**	0.07**	0.23**	-3.00**	0.74**
$ILS_1 \times ILW_2$	-2.20**	-0.02**	-1.00**	0.17**	-1.00**	-0.47**
$ILS_1 \times ILW_3$	1.17**	-0.06**	0.93**	-0.40**	4.00**	-0.27**
$ILS_2 \times ILW_1$	3.78**	0.04**	0.61**	0.06**	2.11**	0.46**
$ILS_2 \times ILW_2$	-1.04**	-0.03**	-0.21**	0.02	-2.89**	-0.17**
$ILS_2 \times ILW_3$	-2.74**	-0.01**	-0.41**	-0.07**	0.78**	-0.29**
$ILS_3 \times ILW_1$	0.43**	-0.01**	-0.26**	0.06**	0.44**	0.60**
$ILS_3 \times ILW_2$	-0.81**	-0.04**	0.49**	-0.21**	0.61**	-0.28**
$ILS_3 \times ILW_3$	0.38**	0.05**	-0.23**	0.15**	-1.05**	-0.32**
$ILS_4 \times ILW_1$	-0.58**	0.01**	-0.14**	-0.02*	-4.50**	-0.43**
$ILS_4 \times ILW_2$	0.13*	-0.02**	0.46**	0.03*	5.00**	0.61**
$ILS_4 \times ILW_3$	0.45**	3.9×10^{-3}	-0.32**	-0.01	-0.50**	-0.18**
$ILS_5 \times ILW_1$	0.62**	1.1×10^{-3}	-0.37**	0.72**	-1.66**	0.49**
$ILS_5 \times ILW_2$	-0.46**	-0.02**	0.40**	-0.45**	2.16**	-0.05**
$ILS_5 \times ILW_3$	-0.16*	0.02**	-0.03	-0.27**	-0.50**	-0.43**
$ILS_6 \times ILW_1$	-4.66**	-0.20**	-0.54**	-0.67**	-2.33**	-0.88**
$ILS_6 \times ILW_2$	2.99**	0.11**	0.48**	0.32**	3.83**	0.16**
$ILS_6 \times ILW_3$	1.67**	0.09**	0.06**	0.35**	-1.50**	0.73**
$ILS_7 \times ILW_1$	-0.09	0.06**	0.61**	-0.11**	0.11	-0.68**
$ILS_7 \times ILW_2$	1.14**	-0.05**	-0.26**	-0.23**	-2.39**	0.03
$ILS_7 \times ILW_3$	-1.04**	-0.01**	-0.35**	0.34**	2.28**	0.65**
$ILS_8 \times ILW_1$	-0.53**	0.02**	0.03*	-0.25**	8.83**	-0.29**
$ILS_8 \times ILW_2$	0.26**	0.06**	-0.36**	0.35**	-5.33**	0.17**
$ILS_8 \times ILW_3$	0.27**	-0.09**	0.33**	-0.10**	-3.50**	0.12**

* and ** SCA estimates are significantly different from zero at >SE and >2SE, respectively.
Hey husked ear yield; Hed husked ear diameter; Hel husked ear length; WSS water soluble solid; Ph plant height; Hd harvest date.

Table S4 Specific combining ability (SCA) for carotenoids of sweet-waxy corn hybrids evaluated across two seasons between 2021 and 2022.

Hybrids	TCC	Lut	Zea	β-Car	β-Cry	α-Xan	β-Cry+Zea	β-Car/β-Cry	β-Cry/Zea	β-Car/(β -Cry+ Zea)
$ILS_1 \times ILW_1$	0.27**	-0.14**	0.36**	0.06**	0.07**	0.22**	0.43**	-0.17**	$2.5x10^{-3}$*	0.10**
$ILS_1 \times ILW_2$	0.05*	-0.09**	-0.26**	-0.04**	-0.05**	-0.35**	-0.30**	-0.20**	$1.6x10^{-3}$	-0.14**
$ILS_1 \times ILW_3$	-0.32**	0.23**	-0.10**	-0.03**	-0.02**	0.13**	-0.13**	0.37**	$1.0x10^{-3}$	0.03**
$ILS_2 \times ILW_1$	0.38**	-0.04**	0.69**	0.15**	0.18**	0.65**	0.84**	-0.88**	0.01**	0.17**
$ILS_2 \times ILW_2$	-0.54**	-0.25**	-0.34**	-0.05**	-0.17**	-0.59**	-0.39**	0.53**	-0.01**	-0.26**
$ILS_2 \times ILW_3$	0.16**	0.29**	-0.35**	-0.09**	-0.01	-0.07**	-0.45**	0.35**	$4.2x10^{-3}$**	0.09**
$ILS_3 \times ILW_1$	0.66**	0.38**	-0.22**	0.14**	-0.42**	0.16**	-0.08**	1.90**	-0.01**	-0.57**
$ILS_3 \times ILW_2$	-0.25**	-0.34**	0.17**	-0.05**	0.34**	-0.17**	0.12**	-0.27**	$4.4x10^{-3}$**	0.63**
$ILS_3 \times ILW_3$	-0.41**	-0.05**	0.05**	-0.09**	0.07**	0.01	-0.03*	-1.63**	$1.0x10^{-3}$	-0.05**
$ILS_4 \times ILW_1$	0.07**	0.21**	-0.30**	-0.04**	-0.08**	-0.09**	-0.34**	0.53**	-0.03**	-0.21**
$ILS_4 \times ILW_2$	-0.05*	0.03**	0.18**	$2.2 x10^{-3}$	0.06**	0.21**	0.18**	-0.06*	0.03**	0.32**
$ILS_4 \times ILW_3$	-0.02	-0.24**	0.12**	0.04**	0.03**	-0.12**	0.16**	-0.47**	$1.2x10^{-3}$	-0.10**
$ILS_5 \times ILW_1$	-0.67**	-0.01	-0.62**	-0.09**	-0.27**	-0.63**	-0.72**	0.65**	-0.01**	-0.33**
$ILS_5 \times ILW_2$	0.20**	-0.17**	0.29**	0.08**	0.05**	0.11**	0.37**	0.48**	0.03**	0.19**
$ILS_5 \times ILW_3$	0.47**	0.18**	0.34**	0.01**	0.22**	0.52**	0.35**	-1.13**	-0.02**	0.14**
$ILS_6 \times ILW_1$	-1.39**	-0.23**	-0.93**	-0.19**	0.30**	-1.17**	-1.12**	-0.44**	0.08**	0.58**
$ILS_6 \times ILW_2$	0.95**	0.42**	0.47**	0.08**	-0.02*	0.80**	0.54**	-0.57**	-0.06**	-0.34**
$ILS_6 \times ILW_3$	0.44**	-0.19**	0.47**	0.11**	-0.28**	0.28**	0.57**	1.01**	-0.02**	-0.24**
$ILS_7 \times ILW_1$	-0.35**	-0.28**	0.33**	-0.02**	0.13**	0.05**	0.31**	-1.07**	$1.0x10^{-4}$	0.18**
$ILS_7 \times ILW_2$	0.09**	0.32**	-0.18**	-0.09**	-0.11**	0.14**	-0.27**	-0.34**	-0.03**	-0.26**
$ILS_7 \times ILW_3$	0.26**	-0.04**	-0.16**	0.11**	-0.01*	-0.20**	-0.04**	1.41**	0.03**	0.08**
$ILS_8 \times ILW_1$	1.04**	0.11**	0.69**	-0.01**	0.10**	0.89**	0.68**	-0.53**	-0.04**	0.09**
$ILS_8 \times ILW_2$	-0.45**	0.07**	-0.33**	0.08**	-0.11**	-0.26**	-0.25**	0.44**	0.04**	-0.14**
$ILS_8 \times ILW_3$	-0.59**	-0.18**	-0.36**	-0.06**	0.01	-0.54**	-0.42**	0.10**	$2.5x10^{-3*}$	0.05**

* and ** SCA estimates are significantly different from zero at >SE and >2SE, respectively.

TCC total carotenoid content; Lut lutein; Zea zeaxanthin; β-Car β-carotene; β-Cry β-cryptoxanthin; α-Xan α-xanthophyll.

Table S5 Magnitude of heterosis over mid-parent (mpH) and better-parent (bpH) for yield-related traits of sweet-waxy corn hybrids evaluated across two seasons between 2021 and 2022.

Hybrids	Hey		Hed		Hel		WSS		Ph		Hd	
	mpH	bpH	mpH	bpH	mpH	bpH	mpH	bpH	mpH	bpH	mpH	bpH
ILS1×ILW1	227.77**	202.89**	41.41**	29.85**	55.93**	52.74**	-4.17*	-6.87*	49.46**	25.10**	-4.80**	-6.51**
ILS1×ILW2	147.99**	142.30**	25.08**	19.32**	38.55**	33.54**	-1.10	-9.11**	50.08**	41.36**	-7.32**	-10.09**
ILS1×ILW3	150.50**	110.73**	27.19**	27.01**	41.13**	28.48**	-4.05*	-13.37**	62.18**	46.77**	-5.80**	-8.90**
ILS2×ILW1	204.76**	141.89**	36.59**	24.27**	49.97**	41.30**	-9.82**	-18.12**	59.30**	34.59**	-4.65**	-6.08**
ILS2×ILW2	123.93**	81.43**	21.56**	14.85**	35.95**	30.13**	-7.17**	-19.95**	49.32**	42.19**	-6.21**	-8.75**
ILS2×ILW3	80.57**	76.31**	25.35**	23.91**	23.88**	21.87**	-6.12**	-20.36**	59.12**	45.51**	-5.23**	-8.08**
ILS3×ILW1	151.20**	100.52**	34.75**	18.73**	42.12**	34.46**	-11.36**	-14.17**	50.57**	30.80**	-2.03*	-3.60*
ILS3×ILW2	113.18**	73.75**	21.10**	10.64**	39.62**	34.20**	-10.52**	-18.05**	48.24**	45.78**	-3.98**	-6.67**
ILS3×ILW3	99.83**	96.60**	26.69**	20.90**	23.74**	21.22**	-5.47**	-14.94**	49.19**	40.68**	-2.86*	-5.87**
ILS4×ILW1	101.05**	41.90*	31.93**	14.96**	35.18**	15.23**	-10.49**	-16.06**	24.11*	18.25*	-7.33**	-9.08**
ILS4×ILW2	90.23**	36.47	18.75**	7.24*	32.11**	14.20*	-7.01**	-17.35**	31.53**	11.17	-6.19**	-9.08**
ILS4×ILW3	75.54**	46.38*	22.55**	15.53**	18.09*	7.68	-5.45**	-17.38**	27.88**	12.13	-6.31**	-9.48**
ILS5×ILW1	157.49**	104.71**	34.68**	20.40**	44.55**	31.95**	-1.56	-5.38*	46.92**	25.53**	-3.42**	-5.88**
ILS5×ILW2	121.46**	79.74**	21.15**	12.38**	42.25**	31.84**	-7.78**	-16.12**	50.25**	44.95**	-4.82**	-8.37**
ILS5×ILW3	97.98**	93.75**	25.68**	21.86**	28.72**	26.46**	-4.39*	-14.54**	49.49**	38.40**	-4.26**	-8.11**
ILS6×ILW1	71.66*	36.48	19.59**	-0.79	35.08**	24.17**	-22.31**	-28.80**	49.43**	31.23**	-8.59**	-10.23**
ILS6×ILW2	140.49**	95.19**	16.09**	-0.43	34.68**	25.71**	-11.98**	-23.43**	56.95**	56.30**	-7.46**	-10.23**
ILS6×ILW3	97.93**	93.70**	18.87**	6.13	21.57**	20.34**	-10.00**	-22.99**	51.67**	44.75**	-5.34**	-8.45**
ILS7×ILW1	190.80**	150.53**	39.42**	23.25**	47.33**	35.86**	-10.13**	-12.96**	42.72**	31.43**	-6.48**	-7.42**
ILS7×ILW2	180.19**	147.09**	23.52**	13.24**	33.33**	24.86**	-7.90**	-15.63**	35.18**	28.83**	-5.87**	-7.96**
ILS7×ILW3	116.44**	100.49**	27.54**	22.15**	22.44**	21.63**	-1.03	-10.93**	46.19**	45.37**	-3.63**	-6.07**
ILS8×ILW1	256.07**	211.91**	36.64**	20.27**	42.82**	41.68**	-11.26**	-15.93**	69.49**	42.41**	-6.57**	-6.67**
ILS8×ILW2	231.21**	175.55**	25.12**	14.19**	31.44**	30.31**	-3.15*	-13.09**	45.04**	37.21**	-6.37**	-7.63**
ILS8×ILW3	166.89**	91.13**	24.25**	18.43**	25.38**	17.20**	-5.03**	-16.23**	52.02**	38.14**	-5.24**	-6.80**

* and ** heterosis estimates are significantly different from zero at >SE and >2SE, respectively.

Hey husked ear yield; Hed husked ear diameter; Hel husked ear length; WSS water soluble solid; Ph plant height; Hd harvest date.

Table S6 Magnitude of heterosis over mid-parent (mpH) and better-parent (bpH) for carotenoids of sweet-waxy corn hybrids evaluated across two seasons between 2021 and 2022.

Hybrids	TCC		Lut		Zea		β-Car		β-Cry		α-Xan		β-Car/β-Cry		β-Cry+Zea		β-Cry/Zea		β-Car/(β -Cry+ Zea)	
	mpH	bpH	mpH	bpH	mpH	bpH	mpH	bpH	mpH	bpH	mpH	bpH	mpH	bpH	mpH	bpH	mpH	bpH	mpH	bpH
$ILS_1 \times ILW_1$	14.39**	5.93	-15.26	-34.14**	34.84**	24.17**	21.02**	7.29**	-67.36**	-70.73**	17.95**	13.45**	221.74**	175.56**	31.41**	27.20**	-15.53	-22.54*	-80.86**	-83.69**
$ILS_1 \times ILW_2$	7.62*	5.82	72.53**	33.90**	-49.99**	-61.96**	-3.25	-13.14**	-28.22**	-37.23**	-3.25	-10.79**	25.90	18.41	-38.91**	-51.78**	96.49**	56.83**	105.69**	30.83
$ILS_1 \times ILW_3$	-5.59	-20.72**	-1.36	-11.11	-21.74**	-44.93**	14.81**	11.80**	-69.47**	-72.58**	-14.66**	-30.99**	206.77**	187.30**	-11.99*	-33.11**	7.29	-15.06	-74.25**	-80.36**
$ILS_2 \times ILW_1$	30.83**	12.16**	5.81	-25.71**	88.32**	65.45**	76.96**	19.89**	-56.48**	-63.69**	58.29**	27.41**	263.47**	118.19**	85.60**	52.30**	4.70	-23.29*	-83.48**	-88.67**
$ILS_2 \times ILW_2$	11.75**	4.36	84.19**	29.17**	-29.15**	-35.60**	46.62**	15.63**	-47.93**	-57.54**	18.76**	5.82	171.14**	78.86**	-12.33*	-13.91*	161.48**	153.10**	-21.09	-54.87**
$ILS_2 \times ILW_3$	22.45**	10.82*	23.40*	-2.22	1.24	-17.02*	58.32**	17.52**	-70.26**	-75.15**	9.85*	6.28	328.22**	166.70**	16.19*	8.43	27.98*	-15.18	-79.50**	-79.55**
$ILS_3 \times ILW_1$	5.31	2.95	39.95**	29.91**	4.15	-7.65	28.18**	9.98**	-76.58**	-80.63**	19.48**	8.01*	363.96**	252.46**	10.90	-2.58	-6.63	-19.07	-89.11**	-93.33**
$ILS_3 \times ILW_2$	-12.46**	-21.80**	72.35**	59.70**	-63.08**	-66.76**	-5.97	-12.60**	55.69**	25.94*	3.10	1.28	-40.29	-45.46*	-47.16**	-52.01**	80.98**	23.50*	196.11**	60.97**
$ILS_3 \times ILW_3$	-23.47**	-40.58**	11.04	0.31	-42.21**	-53.03**	7.11	5.89*	-15.79	-30.22**	-17.28**	-29.46**	4.78	-14.29	-26.54**	-36.31**	12.88	10.27	-23.82	-38.68*
$ILS_4 \times ILW_1$	-3.11	-9.31*	20.98*	10.96	13.76	-7.56	8.73	-7.78**	-74.02**	-77.80**	16.98**	0.16	260.66**	179.64**	12.26*	-7.63	-21.44	-34.55**	-89.45**	-93.67**
$ILS_4 \times ILW_2$	-9.80**	-12.31**	83.00**	67.56**	-53.07**	-53.29**	-1.96	-7.68**	-7.83	-23.04*	16.85**	11.81**	-5.54	-11.36	-37.80**	-39.12**	74.66**	16.26	76.09**	-5.11
$ILS_4 \times ILW_3$	-17.58**	-31.43**	-13.17	-20.63**	-25.24**	-33.68**	20.74**	17.75**	-53.59**	-60.26**	-19.25**	-27.30**	115.62**	80.52**	-9.48	-15.80*	5.66	3.14	-67.06**	-74.58**
$ILS_5 \times ILW_1$	-30.38**	-37.42**	17.01	11.24	-16.62**	-26.07**	-10.21*	-18.76**	-71.56**	-73.55**	-2.02	-10.52**	180.72**	150.08**	-14.75**	-23.98**	-7.16	-16.59	-75.72**	-82.30**
$ILS_5 \times ILW_2$	-24.09**	-37.12**	77.33**	68.27**	-62.92**	-66.61**	-8.30	-19.31**	25.82*	13.96	6.45	3.43	-28.68	-35.69	-46.92**	-52.53**	95.98**	36.82**	270.83**	118.23**
$ILS_5 \times ILW_3$	-28.39**	-47.66**	22.42*	8.14	-32.59**	-45.20**	0.07	-4.68	-0.98	-7.76	-6.45	-20.95**	-10.82	-12.73	-21.76**	-33.15**	9.28	2.61	7.98	-2.38
$ILS_6 \times ILW_1$	-13.10**	-14.44**	-2.94	-10.63	-13.36	-15.05	-9.12*	-9.99**	1.44	-3.73	-9.19*	-13.22**	143.09**	126.19**	-12.12*	-13.09*	29.79*	21.25*	221.52**	193.59**
$ILS_6 \times ILW_2$	20.21**	11.18*	103.34**	86.90**	-31.33**	-43.54**	2.42	-17.89**	18.19	9.19	29.97**	20.58**	-26.69	-43.52*	-21.38**	-35.85**	91.21**	50.35**	247.66**	155.15**
$ILS_6 \times ILW_3$	7.30*	-14.31**	-10.62	-18.61*	-4.58	-28.27**	19.24**	2.47	-65.22**	-66.93**	-7.18	-24.55**	178.69**	137.31**	3.26	-19.04**	16.39	-6.45	-70.87**	-80.99**
$ILS_7 \times ILW_1$	0.13	-5.93	-23.70*	-30.77**	41.39**	31.57**	7.21	0.05	-54.30**	-57.04**	13.77**	4.73	118.90**	90.65**	31.56**	22.53**	-11.84	-20.10*	-64.13*	-72.96**
$ILS_7 \times ILW_2$	2.97	-11.24*	61.91**	47.16**	-38.98**	-53.22**	-11.90*	-32.47**	-26.53*	-29.01**	8.72*	-9.99**	12.17	-17.78	-31.20**	-47.26**	90.11**	53.23**	157.33**	126.68**
$ILS_7 \times ILW_3$	0.60	-24.11**	-22.97*	-39.81**	-15.14*	-39.89**	20.76**	-1.29	-65.47**	-67.60**	-18.70**	-39.85**	205.72**	145.46**	-3.77	-28.82**	20.71	-5.34	-72.65**	-83.76
$ILS_8 \times ILW_1$	8.74*	2.44	-10.89	-23.89**	80.64**	51.43**	18.74**	-13.25**	-61.95**	-62.09**	42.05**	20.01**	224.47**	122.70**	64.48**	32.77**	0.47	-34.31**	-79.33**	-82.68**
$ILS_8 \times ILW_2$	-20.51**	-23.24**	63.55**	39.46**	-46.29**	-48.56**	18.29**	3.49	-30.86**	-33.03**	7.07	0.90	82.33*	45.26*	-30.04**	-30.24**	262.27**	193.25**	104.62**	29.04
$ILS_8 \times ILW_3$	-33.53**	-45.01**	-44.14**	-44.81**	-16.60*	-28.47**	16.59**	-5.01*	-65.24**	-65.29**	-29.36**	-35.48**	214.81**	129.25**	-6.84	-11.30	43.73**	-12.66	-67.93**	-75.19**

* and ** Heterosis estimates are significantly different from zero at >SE and >2SE, respectively.
TCC total carotenoid content; Lut lutein; Zea zeaxanthin; β-Car β-carotene; β-Cry β-cryptoxanthin; α-Xan α-xanthophyll.

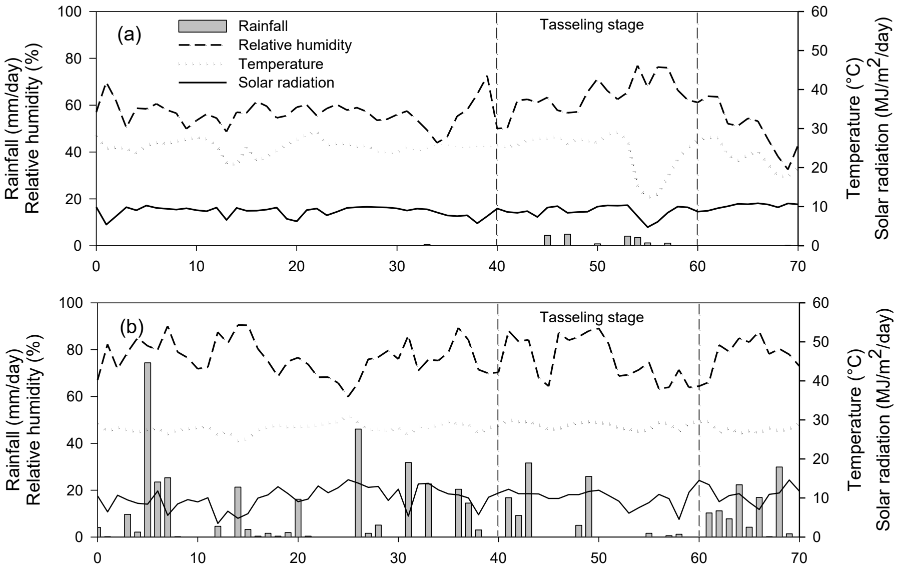

Figure S1. Total rainfall, relative humidity, temperature, and solar radiation during crop growth at the Experimental Field, Thammasat University, Thailand in the dry season of 2021/22 (a) and the rainy season of 2022 (b).
